# CLCA1 Regulates Airway Mucus Production and Ion Secretion Through TMEM16A

**DOI:** 10.3390/ijms22105133

**Published:** 2021-05-12

**Authors:** Raquel Centeio, Jiraporn Ousingsawat, Rainer Schreiber, Karl Kunzelmann

**Affiliations:** Physiological Institute, University of Regensburg, University Street 31, D-93053 Regensburg, Germany; raquel.martins-centeio@vkl.uni-regensburg.de (R.C.); jiraporn.ousingsawat@vkl.uni-regensburg.de (J.O.); rainer.schreiber@vkl.uni-regensburg.de (R.S.)

**Keywords:** CLCA1, TMEM16A, anoctamin 1, airway epithelium, mucus, MUC5AC, KCNN4

## Abstract

TMEM16A, a Ca^2+^-activated chloride channel (CaCC), and its regulator, CLCA1, are associated with inflammatory airway disease and goblet cell metaplasia. CLCA1 is a secreted protein with protease activity that was demonstrated to enhance membrane expression of TMEM16A. Expression of CLCA1 is particularly enhanced in goblet cell metaplasia and is associated with various lung diseases. However, mice lacking expression of CLCA1 showed the same degree of mucous cell metaplasia and airway hyperreactivity as asthmatic wild-type mice. To gain more insight into the role of CLCA1, we applied secreted N-CLCA1, produced in vitro, to mice in vivo using intratracheal instillation. We observed no obvious upregulation of TMEM16A membrane expression by CLCA1 and no differences in ATP-induced short circuit currents (Iscs). However, intraluminal mucus accumulation was observed by treatment with N-CLCA1 that was not seen in control animals. The effects of N-CLCA1 were augmented in ovalbumin-sensitized mice. Mucus production induced by N-CLCA1 in polarized BCi-NS1 human airway epithelial cells was dependent on TMEM16A expression. IL-13 upregulated expression of CLCA1 and enhanced mucus production, however, without enhancing purinergic activation of Isc. In contrast to polarized airway epithelial cells and mouse airways, which express very low levels of TMEM16A, nonpolarized airway cells express large amounts of TMEM16A protein and show strong CaCC. The present data show an only limited contribution of TMEM16A to airway ion secretion but suggest a significant role of both CLCA1 and TMEM16A for airway mucus secretion.

## 1. Introduction

TMEM16A is a Ca^2+^-activated chloride (Cl^−^) channel (CaCC) that belongs to a family of 10 proteins, operating as phospholipid scramblases and Cl^−^ channels [1]. In the lungs, TMEM16A is expressed at low levels in airway epithelial cells and airway and pulmonary arterial smooth muscle cells, and is upregulated during inflammatory airway diseases such as asthma and cystic fibrosis (CF) [2,3,4,5]. However, it remains unclear whether TMEM16A is particularly relevant for mucus production and mucus secretion or for secretion of electrolytes covering the airways (ASL) [5,6,7]. Earlier reports describe that TMEM16A is essential for the function of CFTR by providing local Ca^2+^ levels required for insertion of CFTR into the plasma membrane, and for full activation of CFTR through Ca^2+^-sensitive adenylate cyclase 1 [8,9,10,11,12,13].

Many studies associate expression of TMEM16A and the regulator of Ca^2+^-activated Cl^−^ channels, CLCA1, with goblet cell metaplasia and mucus production/secretion [3,4,5,14,15,16,17]. Inflammatory airway disease is associated with enhanced expression of CLCA1 in airway epithelial cells and high levels in the bronchoalveolar lavage (BAL) fluid [18,19]. It should be mentioned that, different than previously thought, CLCA1 is a secreted protein and a regulator of TMEM16A expression [20]. The so called von Willebrand factor type A (VWA) domain in secreted N-terminal CLCA1 was shown to serve as the minimal requirement for interaction and activation of TMEM16A [21,22]. Interestingly, the microtubular inhibitor nocodazole also augmented TMEM16A currents within minutes after exposure.

Expression of CLCA1 is enhanced in goblet cell metaplasia and associated with various lung diseases [17]. However, mice lacking expression of CLCA1 (mClca3 according to the earlier nomenclature) showed the same degree of mucous cell metaplasia and airway hyperreactivity as wild-type mice when challenged with viruses or allergens [23]. As CLCA1 is not essential for the development of mucous cell metaplasia, expression of other CLCA paralogs was proposed as a mechanism for functional redundancy. Using CLCA1-knockout mice, Mundhenk et al. found that CLCA1 does not contribute to basal, cAMP and Ca^2+^-regulated short circuit currents in mouse trachea. Notably, tracheal instillation of IL-13 in wt mice produced a strong upregulation of CLCA1, along with goblet cell metaplasia and mucin expression, but did not increase Ca^2+^-activated short circuit currents (Isc) [24]. In CLCA1−/− tracheas, goblet cell metaplasia, mucin expression and Ca^2+^-activated Isc were similar, and no compensatory upregulation of CLCA5 was observed.

The controversies regarding the cellular function of CLCA1 triggered the present study, in which we examined the effects of secreted CLCA1 (N-CLCA1) in mouse airways in vivo and in airway epithelial cells in vitro. We found that CLCA1 augments intraluminal mucus, possibly by enhanced mucus production/secretion or by inducing mucus expansion. However, no clear increase in TMEM16A-dependent Cl^−^ transport could be identified.

## 2. Results

### 2.1. Asthmatic Conditions but Not CLCA1 Enhanced Expression of TMEM16A

**Figure 1:**
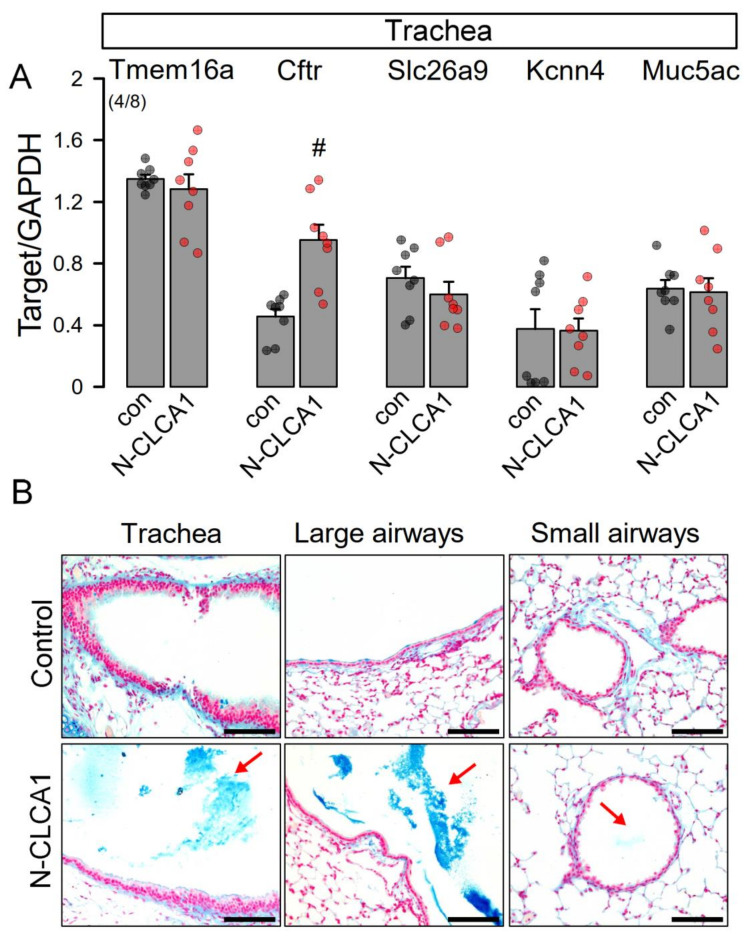
N-CLCA1 increases mucus production in vivo. (**A**) Semiquantitative RT-PCR analysis of the expression of MUC5AC, TMEM16A, CFTR, SLC26A9 and KCNN4 in isolated tracheal epithelial cells from control mice and mice treated for 24 h with N-CLCA1. Mean ± SEM (number of animals/number of reactions). ^#^—significant difference when compared to control or mock (*p* < 0.05; unpaired *t*-test). (**B**) Alcian blue staining indicating enhanced mucus secretion in airways exposed to N-CLCA1. Bar = 100 µm.

CLCA1 has been proposed to augment mucus production and to increase membrane expression of TMEM16A. However, available data are controversial and data from naïve airways are scarce. We therefore exposed mice in vivo to secreted N-terminal CLCA1 protein, which was produced by expression of CLCA1 in HEK293 cells and harvesting of N-CLCA1 from the supernatant (Figure S1). N-CLCA1 or control buffer were applied to mouse airways by intratracheal instillation and animals were sacrificed 24 h later. Airway epithelial cells were harvested from isolated tracheas and expression of secretory ion channels and transporters such as Tmem16A, Cftr, Slc26a9, Kcnn4, as well as Muc5ac was analyzed. Except for Cftr, mRNA expression was not affected by N-CLCA1 (Figure 1A). Our data correspond to a previous report on excised human airway tissue stimulated with inflammatory cytokines in vitro [19]. Moreover, expression of TMEM16A was not detected in mock treated mouse trachea and only occasionally found in mucus producing epithelial cells by immunohistochemistry of N-CLCA1 tracheas (data not shown). Analysis of different airway sections demonstrated an impressive accumulation of mucus in the airways of N-CLCA1 treated animals, suggesting induction of mucus secretion by N-CLCA1 (Figure 1B). In contrast to N-CLCA1-induced secretion of mucus (lower panels), mucus was not detected in airways of mice treated with control supernatant (upper panels).

**Figure 2:**
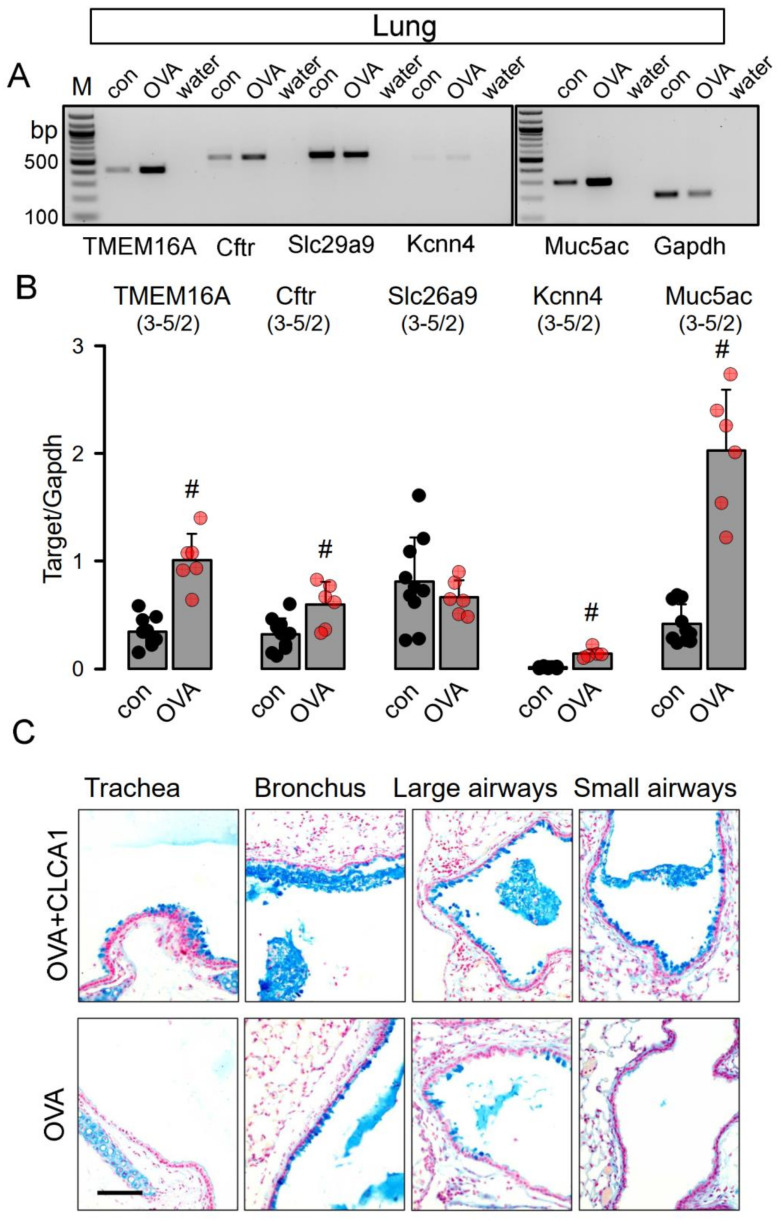
N-CLCA1 enhances mucus secretion in asthmatic mouse lungs in vivo. (**A**,**B**) Semiquantitative RT-PCR analysis of the expression of Tmem16a, Cftr, Slc26a9 and Kcnn4, and Muc5ac in lungs from control mice and OVA-treated mice. Expression of Tmem16a, Cftr, Kcnn4, and Muc5ac is enhanced in asthmatic (OVA) mice. Mean ± SEM (number of animals/number of experiments). ^#^—significant difference when compared to control (*p* < 0.05; unpaired *t*-test). (**C**) Alcian blue staining of airways from OVA-sensitized mice treated with N-CLCA1 (upper panel) and OVA-sensitized mice without additional treatment with N-CLCA1 (lower panel). N-CLCA1 induced additional secretion of mucus with enhanced accumulation of the mucus in central and peripheral airways. Bar = 100 µm.

We also analyzed smaller airways from asthmatic mice sensitized in vivo towards ovalbumin. In contrast to treatment with N-CLCA1, transcriptional upregulation was observed of Tmem16a, Cftr and Muc5ac in airway epithelial cells of asthmatic airways, along with a small but significant increase in Kcnn4 expression (Figure 2A,B). Alcian blue staining indicated pronounced goblet cell metaplasia in airways of asthmatic mice [6] (Figure 2C, OVA, lower panel). Additional intratracheal application of N-CLCA1 in OVA-sensitized asthmatic mice further augmented goblet cell metaplasia and induced mucus secretion with intraluminal accumulation in the airways. These effects of N-CLCA1 may be related to the mucus expanding properties of CLCA1 described earlier [25]. However, as previous studies demonstrated a role of TMEM16A for mucus secretion, N-CLCA1 may also induce mucus secretion through an increase in TMEM16A-function [5,16,21].

### 2.2. KCNN4 Is Upregulated in Asthma Which Counteracts Activation of TMEM16A

**Figure 3:**
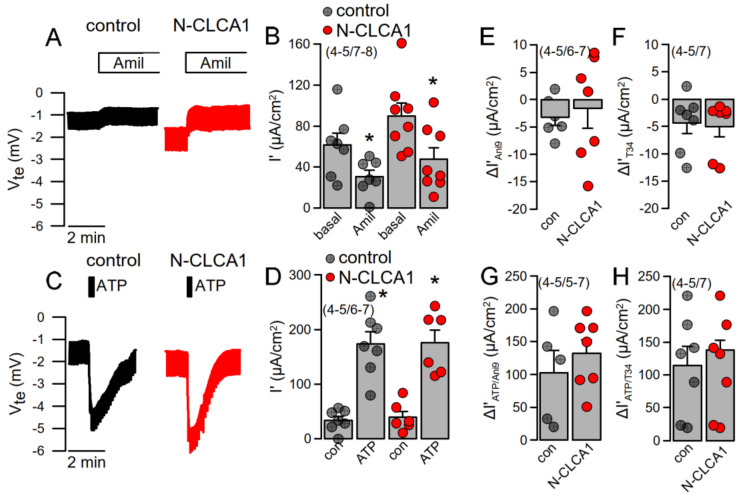
N-CLCA1 does not induce additional Ca^2+^-activated Cl^−^ secretion in mouse tracheas. (**A**,**B**) Original Ussing chamber recording of the transepithelial voltages measured in mouse trachea under open circuit conditions and summary of the calculated equivalent short circuit current (Isc’). No significant changes in amiloride-sensitive (10 µM) Na^+^ absorption was detected by 24 h exposure to N-CLCA1. (**C**,**D**) No significant changes in ATP (luminal; 100 µM)—induced negative voltage deflection and ATP-activated Isc’ was detected by N-CLCA1. (**E**,**F**) N-CLCA1 did not induce an effect of Ani9 (10 µM) or TRAM-34 (100 nM) on basal currents. (**G**,**H**) Effects of ATP in the presence of luminal Ani9 or basolateral TRAM-34 (T34) were independent of N-CLCA1. Mean ± SEM (number of animals/number of experiments). *—significant effect of amiloride and ATP (*p* < 0.05; paired *t*-test).

Ion transport was assessed under open circuit conditions in tracheas of control mice and mice treated with N-CLCA1. No differences were found in basal amiloride-sensitive Na^+^ absorption (Figure 3A,B). Moreover, ATP-activated ion transport was not different in N-CLCA1-treated mice when compared to control mice (Figure 3C,D). In the presence of the TMEM16A-blocker Ani9 or the KCNN4 inhibitor TRAM-34, both basal and ATP-activated transport were similar in CLCA1 and control treated airways (Figure 3E–H). Interestingly, we detected an upregulation of KCNN4 in tracheas and other airway sections of asthmatic (OVA-treated) mice (Figure 2A, Figure S2A–C). Moreover, KCNN4 was also upregulated by IL-13 in Calu3 airway epithelial cells (Figure S2D,E). We examined whether both Ca^2+^-activated KCNN4 K^+^ channels and TMEM16A Cl^−^ channels are activated by increase in intracellular Ca^2+^ with ATP using patch clamp experiments, as this question cannot be clearly examined in Ussing chamber recordings. In fact, the data demonstrate, that KCNN4 is activated by increase in intracellular Ca^2+^, which corresponds to earlier observation by Lee and Fosket.

Under control conditions, ATP activated only small ion currents in Calu3 cells. In contrast, ATP hyperpolarized the membrane voltage and clearly activated a whole cell K^+^ current in cells exposed to IL-13 (Figure S2F,G). Inhibition of KCNN4 by TRAM-34 abolished the ATP-induced hyperpolarization and induced a strong outward rectification (Figure S2F–H). Peak currents measured at the clamp voltage of +100 mV were similar in the absence or presence of TRAM-34. In the absence of TRAM-34, whole cell currents were dominated by KCNN4, while TMEM16A currents dominated in the presence of TRAM-34—i.e., in the absence of KCNN4 currents. In the absence of KCNN4 currents cells are depolarized which facilitates activation of voltage-dependent TMEM16A channels by increase in intracellular Ca^2+^. In contrast, in the absence of TRAM-34, Ca^2+^-dependent activation and hyperpolarization by KCNN4 inhibits activation of TMEM16A [26]. This is further supported in the human CF submucosal airway epithelial cell line 6CFSMEo-. This cell line demonstrates pronounced expression of both TMEM16A and KCNN4, but no or little expression of CFTR, CLCA1, and the potassium channel KCNQ1 (Figure S3A). Stimulation by ATP activated a whole cell K^+^ current and hyperpolarized the membrane voltage, which was more pronounced than in Calu3 cells. The KCNN4 inhibitor TRAM-34 abolished hyperpolarization by ATP-dependent activation of KCNN4, but peak currents at +100 mV remained unchanged. The results demonstrate again a predominant activation of Ca^2+^-activated K^+^ currents by ATP inducing pronounced hyperpolarization, which counteracts activation of TMEM16A [26] (Figure S3B,C). In contrast, TMEM16A is activated in the presence of TRAM-34 and the TMEM16A-blocker Ani9 fully inhibited ATP-activated whole cell currents (Figure S3D).

### 2.3. Expression and Plasma Membrane Localization of TMEM16A Is Enhanced by IL-13 and N-CLCA1, Respectively

**Figure 4:**
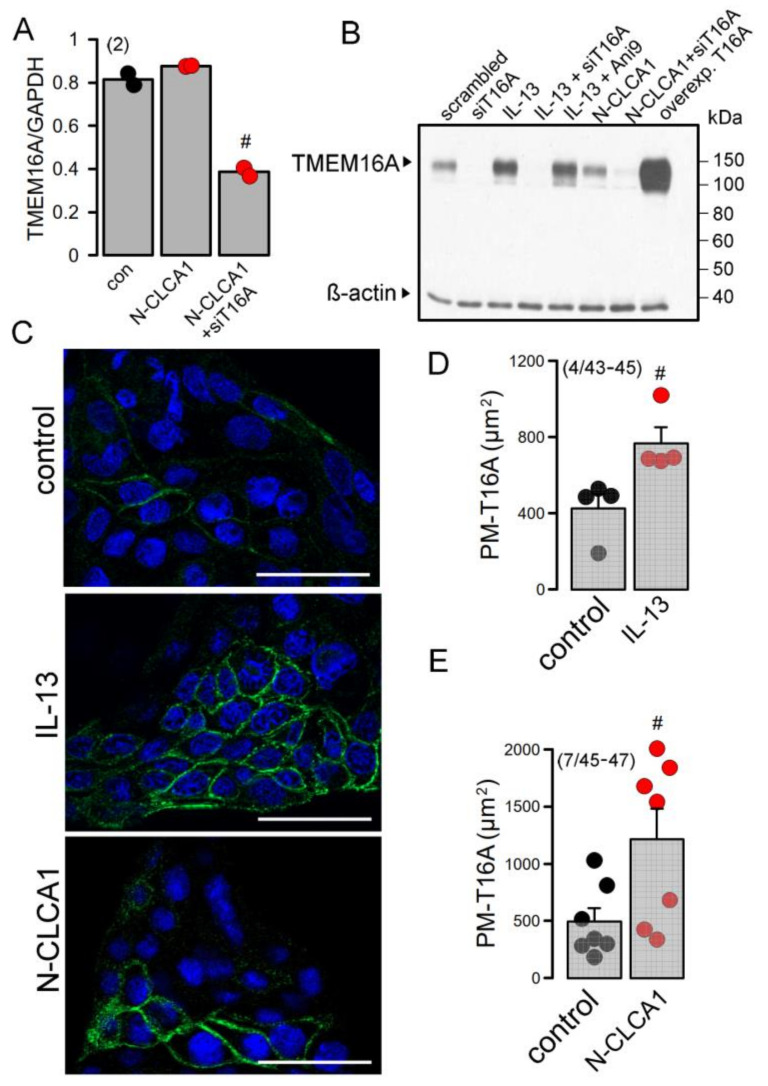
CLCA1 does not enhance expression of TMEM16A but stabilizes TMEM16A in the plasma membrane of Calu3 cells. (number of cover slips/number of cells)y. (**A**) Semiquantitative RT-PCR shows no increase in TMEM16A expression by exposure to N-CLCA1. (**B**) Western blot of TMEM16A indicates increase in expression by IL-13 but not by N-CLCA1. (**C**) Staining of TMEM16A in the plasma membrane indicates low expression in Calu3 cells. Exposure of Calu3 cells to IL-13 (20 ng/mL) increases expression of TMEM16A. N-CLCA1 does not increase the number of Calu3 cells expressing TMEM16A, but rather enhances membrane expression of TMEM16A in some cells. Bars = 50 µm. (**D**) Quantification of TMEM16A plasma membrane staining in the absence or presence of IL-13. (number of cover slips/number of cells). (**E**) Quantification of TMEM16A plasma membrane staining in the absence or presence of CLCA1. (number of cover slips/number of cells). Mean ± SEM ^#^—significant difference when compared to control or control (*p* < 0.05; unpaired *t*-test).

Above data indicate an upregulation of TMEM16A expression under asthmatic conditions and by the cytokine IL-13 [16]. N-CLCA1, in contrast, did not induce expression of TMEM16A in Calu3 cells (Figure 4A). This is further supported by Western blotting of TMEM16A. IL-13 but not by CLCA1 enhanced expression of TMEM16A (Figure 4B). Immunofluorescence staining of TMEM16A demonstrated enhanced expression of TMEM16A upon treatment of Calu3 cells with the Th2-cytokine IL-13. CLCA1 did not increase the number of TMEM16A-positive cells but seemed to enhance membrane expression of TMEM16A (Figure 4C). Quantification of the immunofluorescence in the plasma membrane suggested an increase in plasma-membrane localization of TMEM16A by both IL-13 and N-CLCA1 (Figure 4D,E). We analyzed whole cell currents activated by ATP in the absence and presence of N-CLCA1. In the absence of N-CLCA1 very little current was activated by ATP (Figure S4). Upon exposure to N-CLCA1, cells were hyperpolarized by application of ATP, suggesting activation of KCNN4 K^+^ channels. In the presence of TRAM-34 (T34), ATP no longer hyperpolarized cells and activated a more outwardly rectifying whole cell current, suggesting predominant activation of TMEM16A (Figure S4). Taken together, N-CLCA1 increases KCNN4 currents, possibly by enhancing plasma membrane localization of TMEM16A, which has been shown earlier to increase intracellular Ca^2+^ signals elicited by ATP [27].

### 2.4. TMEM16A Supports Expression of SPDEF, the Regulator of Goblet Cell Metaplasia

**Figure 5:**
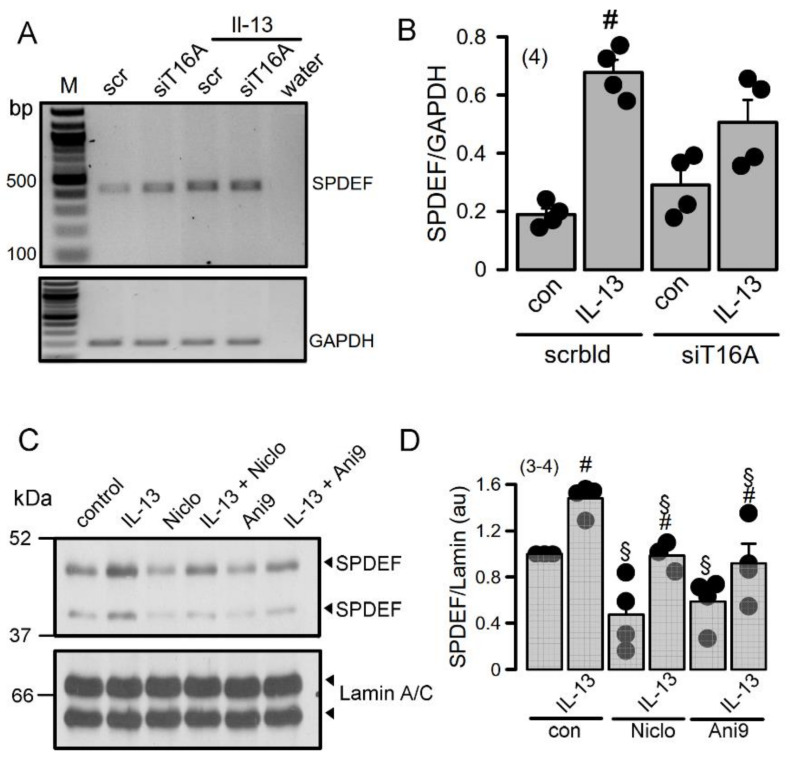
TMEM16A supports upregulation of the master switch for goblet cell metaplasia, SPDEF. (**A**,**B**) RT-PCR analysis suggests inhibition of SPDEF expression by siRNA-knockdown of TMEM16A. (number of experiments). (**C**,**D**) Western blots indicating inhibition of SPDEF expression by inhibitors of TMEM16A. (number of experiments). Mean ± SEM. ^#^—significant difference when compared to scrambled, control, and L-13, respectively (*p* < 0.05; unpaired *t*-test).

RT-PCR analysis of SPDEF expression in Calu3 cells indicated upregulation by incubation with the cytokine IL-13 (Figure 5A,B). siRNA knockdown of TMEM16A inhibited upregulation of SPDEF by IL-13 suggesting a role Of TMEM16A for expression of SPDEF. Similar inhibition of SPDEF expression by siTMEM16A was observed by Western blotting of SPDEF protein (upper and lower bands indicate glycosylated and nonglycosylated protein, respectively) (Figure 5C). Moreover, densitometric quantification of the Western blots also indicated inhibition of SPDEF expression by the TMEM16A inhibitors niclosamide and Ani9 (Figure 5D). Taken together, TMEM16A expression and function controls expression of the master switch for goblet cell metaplasia, SPDEF [28,29,30].

### 2.5. CLCA1-Induced Mucus Production in Polarized BCi-NS1 Cells Is TMEM16A-Dependent

**Figure 6:**
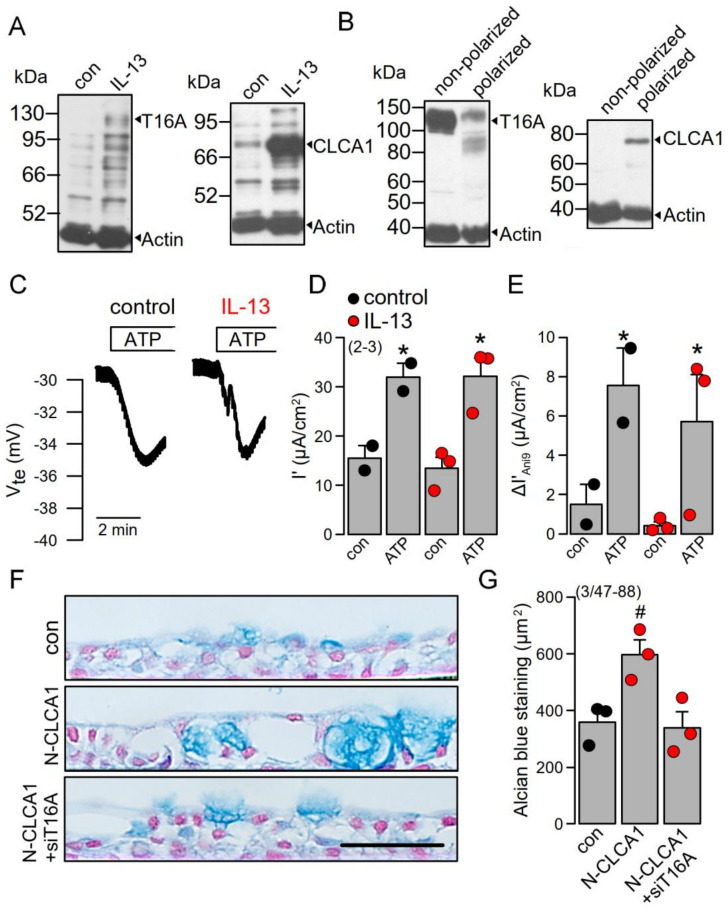
CLCA1-induced mucus production in polarized BCi-NS1 cells is TMEM16A-dependent. **A**,**B**) Western blot indicating upregulation of CLCA1 expression in BCi-NS1 cells exposed to IL-13 (20 ng/mL). Expression of TMEM16A was found to be attenuated in polarized grown cells (filter-grown ALI-cultures), when compared to nonpolarized (plastic-grown) cells. In contrast, CLCA1 expression was upregulated in ALI cultures. (number of experiments). (**C**,**D**) IL-13 does not enhance ATP-induced (100 µM) negative voltage deflections and activation of short circuit currents (Isc). Activation of Isc was fully transient, i.e., the current returned to the baseline. (**E**) The effect of the TMEM16A inhibitor Ani9 (10 µM) is not enhanced in IL-13 treated cells. (**F**,**G**) Individual examples and quantification of alcian blue staining in polarized BCi-NS1 cells under control conditions, after exposure to N-CLCA1 or in cells treated with N-CLCA1 and siTMEM16A. Mucus expression is enhanced in N-CLCA1-treated cells, but the effect of N-CLCA1 is blocked after knockdown of TMEM16A. (number of experiments). Scale bar = 50 µm. Mean ± SEM. ^#^ significant difference when compared to control (*p* < 0.05; unpaired *t*-test). * significant activation by ATP (*p* < 0.05; paired *t*-test).

The present and previous data [16] data suggest an important role of TMEM16A for IL-13 and CLCA1-induced mucus/MUC5AC production. We explored the role of TMEM16A/CLCA1 also in polarized grown BCi-NS1 human airway epithelial cells [16,31]. While expression of both TMEM16A and CLCA1 was very low in these cells, exposure of the cells to IL-13 increased expression of both proteins (Figure 6A). Interestingly, expression of TMEM16A was strongly reduced upon polarization of the cells, while expression of CLCA1 was upregulated (Figure 6B). Ussing chamber recordings under open circuit conditions demonstrated the typical negative voltage deflections upon stimulation with ATP, which were, however, not enhanced by IL-13 (Figure 6C,D). The basal properties of the Bci-NS1 cells were already reported in our previous study. The transepithelial resistance TEER (R_te_) was >2 kVcm^2^ and was not affected by treatment with IL-13. Amiloride sensitive Isc was 9.8 µA/cm^2^ for both control cells and cells stimulated with IL-13. Moreover, ATP-effects in the presence of Ani9 were similar in the absence or presence of IL-13 (Figure 6E). Exposure of the cells to N-CLCA1 upregulated mucus production, which was inhibited by simultaneous knockout of TMEM16A (siT16A, Figure 6F,G). The results obtained in these polarized airway epithelial cells confirm the important role of TMEM16A not only for IL-13-induced goblet cell metaplasia [16], but also demonstrate an important function of TMEM16A in CLCA1-induced mucus production.

## 3. Discussion

The calcium-activated chloride channel regulator 1 (CLCA1) was shown to be most upregulated gene transcript (>100-fold) in a study with diisocyanate-induced asthmatic mice [32]. The ability of diisocyanate to induce CLCA1 was remarkable given the absence of TH2-type T cell cytokines IL-4 or IL-13. Because in this study the pro-asthmatic effects of diisocyanate were inhibited by crofelemer, the authors suggested a role of TMEM16A-upregulation of mucus and CLCA1. A role of TMEM16A for mucus production and mucus secretion was also suggested from our earlier studies [5,16] and a number of other reports [3,4,15,33,34,35]. Additionally, the present data suggest a role of TMEM16A for mucus production induced by CLCA1 in polarized airway epithelial cells (Figure 6). The expression levels of TMEM16A in mouse airways and polarized human airway epithelia are very low [7,12] and its contribution to Ca^2+^-activated Cl^−^ secretion is rather limited (Figure 1 and Figure 6). Low expression of TMEM16A may not be misinterpreted as a missing role in mucus secretion [7]. A number of previous reports demonstrate TMEM16A as a membrane tether of the endoplasmic reticulum and a regulator of spatial sub-membranous Ca^2+^ signals [5,27,36]. Typically, expression of Ca^2+^ -regulating proteins such as IP3 receptors or Ca^2+^ influx channels is scarce in terminally differentiated cells but is often enhanced when cells start proliferating. As described in our previous reports, TMEM16A expression is largely enhanced in proliferating cells [37,38,39]. 

The role of TMEM16A for Ca^2+^-dependent Cl^−^ secretion in the airways may have been overestimated in previous studies. The reduced ATP-induced Cl^−^ secretion observed in the airways of TMEM16A-knockout mice [40,41] is at least in part due to the compromised function of CFTR [8,9,10,11,12,13]. Moreover, the use of nonspecific inhibitors of TMEM16A such as CaCCinhAO1, niclosamide, niflumic acid and others may also contribute to this overestimation. The present study also suggests that Ca^2+^-activated KCNN4 K^+^ channels are upregulated by Th2-dependent stimulation (IL-13, OVA) and are activated by purinergic stimulation, which hyperpolarizes the membrane voltage upon Ca^2+^-dependent stimulation and disables activation of TMEM16A (Figure 2, Figures S2–S4). Activation of KCNN4 is essential to maintain the electrical driving force for Cl^−^ secretion during Ca^2+^—dependent stimulation. Thus, we would disagree with a recent report that claims inhibition of KCNN4 to reduce Na^+^ absorption and to improve mucociliary clearance in patients with cystic fibrosis [42]. In fact, ASL in human airways relies essentially on submucosal gland secretion, which essentially need KCNN4. A number of papers indicate that basolateral Ca^2+^-activated SK4-like chloride channels are required for chloride secretion by the airway epithelium [43] [44,45,46,47]. Moreover, submucosal bicarbonate secretion also essentially requires activation of Ca^2+^-dependent SK4 potassium channels [48,49]. Inhibition of KCNN4 is therefore likely to worsen CF-lung disease.

An important finding of the present study is the impact of TMEM16A expression and the TMEM16A inhibitors niclosamide and Ani9- [14,50] on IL-13-induced expression of SPDEF. Airway epithelial SPDEF is a transcriptional regulator that integrates goblet cell differentiation and pulmonary Th2 inflammation [28,29,30]. This present observation may also help to explain why niclosamide had a clear inhibitory effect on mucus production in our previous study [51].

Accumulation of mucus in N-CLCA1-treated mice was an obvious finding of the present study (Figure 1 and Figure 2). CLCA1 may have enhanced membrane expression of TMEM16A and thereby may have increased mucus secretion. However, CLCA1 may have also contributed to unfolding and expansion of airway mucus, similar to unfolding of intestinal mucus [25,52]. Although CLCA1 was proposed to induce this effect by activating CaCC/TMEM16A in vitro [16,17,18,19,20,21,53], our present in vivo data do not indicate an upregulation of TMEM16A currents in mouse trachea. Thus, our data agree with other in vivo studies that indicate (i) no contribution of CLCA1 chloride conductance in mouse airways [24] and (ii) no inhibition of CLCA1-indued mucus expansion by TMEM16A channel blockers in mouse intestine [25]. Thus, results obtained in vitro may not necessarily reflect the situation in vivo. Along this line, we may refer to our previous observations that indicate a remarkable difference in functional regulation of TMEM16A, when comparing endogenous TMEM16A with overexpressed TMEM16A [54,55]. Data on the regulation of TMEM16A by CLCA1 obtained exclusively in heterologous expression systems may not necessarily apply in vivo. In conclusion, the regulator of TMEM16A, CLCA1, is a potent stimulus for mucus secretion and, surprisingly, for mucus production. However, CLCA1 had no measurable effect on TMEM16A-dependent Cl^−^ transport in naïve mouse airways and differentiated human airway epithelia, probably due to its low expression in fluid secretory cells. We speculate that activation of TMEM16A will probably not compensate for defective Cl^−^ secretion in cystic fibrosis.

## 4. Materials and Methods

### 4.1. Animals and Treatments

Allergen challenge of mice has been described previously [56]. In brief, mice were sensitized to ovalbumin (OVA; Sigma-Aldrich, St. Louis, MO, USA) by intraperitoneal (I.P.) injection of 100 µg OVA in 100 µL Aluminum Hydroxide Gel Adjuvant (InvivoGen, San Diego, CA, USA) on days 0 and 14. At days 21 to 23, mice were anesthetized (ketamine—90–120 mg/kg and xylazine—6–8 mg/kg) and challenged to OVA by intratracheal (I.T.) instillation of 50 µg OVA in 100 µL saline. Control mice were sham-sensitized with aluminum hydroxide gel and challenged to saline by I.T. instillation. Allergen reaction was allowed to develop for 72 h. CLCA1-conditioned media (N-CLCA1; 100 µL; preparation described below) was administered to control or OVA-challenged animals by I.T. instillation 24 h before sacrificing the animals. Control I.T. instillation was performed with mock-conditioned media. All animal experiments complied with the reporting of in vivo experiments guidelines and were carried out in accordance with the United Kingdom Animals Act, 1986, and associated guidelines, and EU Directive 2010/63/EU for animal experiments. All animal experiments were approved by the local Ethics Committee of the Government of Unterfranken/Wurzburg (AZ: 55.2-2532-2-1359-15) and were conducted according to the guidelines of the American Physiologic Society and German Law for the Welfare of Animals.

### 4.2. Cell Culture and Treatments

All cells were grown at 37 °C in a humidified atmosphere with 5% CO_2_. Calu3 human airway epithelial cells were grown in DMEM/Ham’s F-12 with L-Glutamine medium supplemented with 10% (*v/v*) fetal bovine serum (FBS), 1% (*v/v*) L-glutamine 200 mM and 1% (*v/v*) HEPES 1M (all from Capricorn Scientific, Ebsdorfergrund, Germany). Human embryonic kidney HEK293T cells were grown in DMEM low glucose medium supplemented with 10% (*v/v*) FBS and 1% (*v/v*) L-glutamine 200 mM (all from Capricorn Scientific, Ebsdorfergrund, Germany).

Construction of expression plasmids has been described previously [57]. In brief, CLCA1 was cloned from human colon and ligated into a pcDNA3.1 vector. For conditioned media preparation, HEK293T cells were seeded at 500,000 cells per 6 well and next day transfected with empty pcDNA3.1 (mock) or hCLCA1 plasmids using standard protocols for Lipofectamine 3000 (Invitrogen, Carlsbad, CA, USA). After 6 h, the medium was changed to serum-free media (Opti-MEM Reduced Serum Medium (Gibco/Thermo Fisher Scientific, Waltham, MA, USA)) and 24 h later the supernatants—control or enriched in secreted CLCA1 protein (N-CLCA1) were collected. BCi-NS1 cells (kindly provided by Prof. R. Crystal, Weill Cornell Medical College, New York, NY, USA) were cultured in supplemented Bronchial Epithelial Cell Growth Medium (Lonza, Basel, Switzerland). For polarization, BCi-NS1 cells were seeded onto permeable supports with a pore size of 0.4 µm (Snapwell #3801, Corning, New York, NY, USA) in an air–liquid interface by seeding 300,000 cells on human type IV collagen (Sigma-Aldrich, St. Louis, Missouri, USA)—coated inserts. Cells were maintained in a media consisting of 1:1 DMEM:Ham’s F12 supplemented with 10% (*v/v*) FBS, 1% (*v/v*) P/S, 0.5% (*v/v*) Amphotericin B and 0.1% (*v/v*) Gentamycin (all from Capricorn Scientific, Ebsdorfergrund, Germany). On the following day, the serum was replaced with 2% (*v/v*) of serum-substitute Ultroser G (Pall Life Sciences, New York, NY, USA). On the second day after seeding, the media was removed from the upper compartment to expose the apical surface to air and to establish an air–liquid interface (ALI). Cells were grown for 28 days on filters, and transepithelial resistances (TEER) were measured regularly, which was in the range as described earlier [16].

Cells were treated with IL-13 (20 ng/mL; Enzo Life Sciences, Lörrach, Germany) for 72 h in Opti-MEM, and treatment was refreshed every day in the absence or presence of the inhibitors Ani9 and Niclosamide-ethanolamine (both 1 µM; Sigma-Aldrich, St. Louis, Missouri, USA). Treatment with mock- or CLCA1-conditioned media was applied for 24–48 h. Treatment of polarized BCi cells was performed both in the basolateral media and by adding 100 µL to the apical compartment. Knockdown of TMEM16A was performed by transfection of siRNA against TMEM16A (5-GGUUCCCAGCCUACCUCACUAACUU-3; Invitrogen, Carlsbad, California, USA) using standard protocols for Lipofectamine 3000. Scrambled siRNA (Silencer^®^ Select Negative Control siRNA #1, Ambion, Austin, TX, USA) was transfected as negative control. Knockdown of TMEM16A was validated as reported earlier [16]. All experiments were performed 48–72 h after transfection.

### 4.3. RT-PCR

For semiquantitative RT-PCR total RNA from tracheal epithelial cells, lung tissue, Calu3 or 6CFSMEo^−^ cells was isolated using NucleoSpin RNA II columns (Macherey-Nagel, Düren, Germany). Total RNA (0.5 µg/25 µl reaction) was reverse-transcribed using random primers (Promega, Mannheim, Germany) and M-MLV Reverse Transcriptase RNase H Minus (Promega, Mannheim, Germany). Each RT-PCR reaction contained sense (0.5 µM) and antisense primers (0.5 µM) (Table 1), 0.5 µl cDNA and GoTaq Polymerase (Promega, Mannheim, Germany). After 2 min at 95 °C, cDNA was amplified (targets 30–35 cycles, reference Gapdh 25 cycles) for 30 s at 95 °C, 30 s at 56 °C and 1 min at 72 °C. PCR products were visualized by loading on Midori Green Xtra (Nippon Genetics Europe) containing agarose gels and analyzed using Image J 1.52r (NIH, Bethesda, MD, USA).

### 4.4. Transepithelial Ussing Chamber Recordings

Animals were sacrificed by cervical dislocation and isolated tracheas were cleaned, sectioned and kept in ice-cold standard bicarbonate-free Ringer’s solution (in mM: NaCl 145, KH_2_PO_4_ 0.4, K_2_HPO_4_ • 3 H_2_0 1.6, Glucose 5, MgCl_2_ • 6 H_2_0 1, Ca-Gluconate • 1 H_2_0 1.3; pH 7.4). Tissues were mounted into Ussing chambers in supports with a circular aperture of 0.177 cm^2^. Polarized cell inserts were mounted onto supports with a circular aperture of 1.13 cm^2^. Luminal and basolateral sides of the epithelia were perfused continuously at a rate of 5 mL/min with standard bicarbonate-free Ringer’s solution ± compounds (Amiloride 10 µM, apical; Tram34 100 or 300 nM, basolateral; Ani9 10 µM, apical; ATP 100 µM, apical; all from Sigma-Aldrich, St. Louis, Missouri, USA). Luminal and basolateral solutions were heated to 37 °C using a water jacket. Experiments were carried out under open-circuit conditions. Data were continuously collected using PowerLab software (AD Instruments, Spechbach, Germany). Values for transepithelial voltages (V_te_) were referred to the serosal side of the epithelia. Transepithelial resistances (R_te_) were determined by applying short (1 s) current pulses (ΔI = 0.5 µA). Typically, the transepithelial resistance was between 45 and 65 Ωcm^2^ and was not changed by application of CLCA1. R_te_ and equivalent short circuit currents (I’sc) were calculated according to Ohm’s law (R_te_ = ΔV_te_/ΔI, Isc = V_te_/R_te_).

### 4.5. Western Blotting

Protein was isolated from cells using a lysis buffer containing 25 mM Tris-HCl pH 7.4, 150 mM NaCl, 1 mM EDTA, 5% glycerol, 0.43% Nonidet P-40, 100 mM dithiothreitol (both from PanReac AppliChem, Barcelona, Spain) and 1× protease inhibitor mixture (Roche, Basel, Switzerland). For supernatant protein precipitation, the trichloroacetic acid (TCA) method was used. Proteins were then separated by 8.5% SDS-PAGE and transferred to a PVDF membrane (GE Healthcare, Munich, Germany). Membranes were incubated overnight at 4 °C with primary antibodies: rabbit anti-TMEM16A (#ab64085, Abcam, Cambridge, UK; 1:500 in 1% (*w/v*) NFM/TBS-T), rabbit anti-CLCA1 (#ab180851, Abcam; 1:1000 in 3% (*w/v*) NFM/TBST-T), mouse anti-(S)PDEF(G10) (#sc-166846, Santa Cruz Biotechnology; 1:250 in 3% (*w/v*) NFM/TBST-T) and rabbit anti-KCNN4 (#APC-064, Alomone Labs, Jerusalem, Israel; 1:500 in 3% (*w/v*) NFM/TBST-T). The antibodies rabbit anti-Actin (#A2066; Sigma-Aldrich, St. Louis, Missouri, USA; 1:10 000 in 5% (*w/v*) NFM/TBS-T), rabbit anti-Lamin A/C (#sc-20681, Santa Cruz Biotechnology, Dallas, TX, USA; 1:60,000 in 3% (*w/v*) NFM/TBST-T) and rabbit anti-Na+/K+-ATPase (#sc-28800, Santa Cruz Biotechnology; 1:1500 in 5% (*w/v*) NFM/TBST-T) were used as loading controls. Membranes were then incubated with horseradish peroxidase (HRP)-conjugated goat anti-rabbit or sheep anti-mouse secondary antibodies at room temperature for 2 h and immunoreactive signals were visualized using a SuperSignal HRP Chemiluminescence Substrate detection kit (#34577; Thermo Fisher Scientific, Waltham, MA, USA).

### 4.6. Immunocytochemistry

For TMEM16A staining in Calu3 airway cells, cells seeded onto glass coverslips were fixed with a pre-cooled 3:1 mix of methanol:acetone for 10 min at −20 °C, washed in PBS with Ca^2+^ and Mg^2+^ (PBS^++^), blocked with 5% BSA/PBS^++^ for 30 min at room temperature, incubated with rabbit anti-TMEM16A antibody (1:100 in 1%BSA/PBS^++^; #ab64085, Abcam, Cambridge, UK) for 1h at 37 °C, washed, incubated with Alexa Fluor 488-labeled donkey anti-mouse IgG (1:400 in 1%BSA/PBS^++^, Invitrogen, Carlsbad, California, USA) and counterstained with Hoe33342 (1:200) for 1 h at room temperature. Cells were then washed and mounted in fluorescence mounting medium. Plasma membrane (PM) staining was quantified using ImageJ and Zen. The quantitative analysis was performed by measuring the fluorescence intensity in the region of interest (ROI). ROIs are reflected by the plasma membrane and were detected by the analysis software. The settings for excitation and exposure time were identical for all measurements.

### 4.7. Mucus Staining in Polarized Cells

BCi cells polarized in permeable supports were fixed in 4% PFA/PBS for 20 min at room temperature and embedded in paraffin. Paraffin cuts of 5 µm were deparaffinized, stained with standard Alcian blue solution and counterstained with Nuclear Fast Red solution (Sigma-Aldrich, St. Louis, Missouri, USA). After dehydration and clearing steps, sections were mounted in DePeX mounting medium (SERVA Electrophoresis, Heidelberg, Germany). Stains were assessed by light microscopy. Mucus-stained (blue) areas were determined using ImageJ.

### 4.8. Mucus Ctaining in Lungs

Mouse lungs and tracheas were fixed by transcardial perfusion and lung perfusion with 4% paraformaldehyde in PBS. Tissues were incubated overnight in fixative solution and then embedded in paraffin. Paraffin-embedded sections of 5 μm were deparaffinized, stained with standard Alcian blue solution and counterstained with Nuclear Fast Red solution (Sigma-Aldrich, Darmstadt, Germany). After dehydration and clearing steps, whole mouse lungs sections were mounted in DePeX mounting medium (SERVA Electrophoresis, Heidelberg, Germany). Stains were assessed by light microscopy and determined using ImageJ.

### 4.9. Patch Clamp

Cells were patch clamped when grown on coated glass coverslips. Coverslips were mounted in a perfused bath chamber on the stage of an inverted microscope (IM35, Zeiss) and kept at 37 °C. Patch pipettes were filled with a cytosolic-like solution containing (in mM): KCl 30, K-Gluconate 95, NaH_2_PO_4_ 1.2, Na_2_HPO_4_ 4.8, EGTA 1, Ca-Gluconate 0.758, MgCl_2_ 1.03, D-Glucose 5, ATP 3; pH 7.2. The intracellular (pipette) Ca^2+^ activity was 0.1 µM. The bath was perfused continuously with standard bicarbonate-free Ringer’s solution (in mM: NaCl 145, KH_2_PO_4_ 0.4, K_2_HPO_4_ 1.6, Glucose 5, MgCl_2_ 1, Ca-Gluconate 1.3) at a rate of 8 mL/min. Patch pipettes had an input resistance of 2–5 MΩ and whole cell currents were corrected for serial resistance. Currents were recorded using a patch clamp amplifier EPC9, and PULSE software (HEKA, Lambrecht, Germany) as well as Chart software (AD Instruments, Spechbach, Germany). Cells were stimulated with 1 µM ATP in the absence and presence of TRAM34. In regular intervals, membrane voltage (*V*c) was clamped in steps of 20 mV from −100 to +100 mV from a holding voltage of −100 mV. Current density was calculated by dividing whole cell currents by cell capacitance.

### 4.10. Materials and Statistical Analysis

All compounds used were of highest available grade of purity and were bought from Sigma-Aldrich (St. Louis, Missouri, USA), unless indicated otherwise. Data are shown as individual traces/representative images and/or as summaries with mean values ± SEM, with the respective number of experiments given in each figure legend. For statistical analysis, paired or unpaired Student’s *t*-test or ANOVA were used as appropriate. A *p*-value of <0.05 was accepted as a statistically significant difference.

## Figures and Tables

**Table 1:** Primers used for PCR.

Gene Accession Number	Primer	Size (bp)
Mouse Tmem16a NM_001242349.2	s: 5′-GTGACAAGACCTGCAGCTAC as: 5′-GCTGCAGCTGTGGAGATTC	406
Human TMEM16A NM_018043.7	s: 5′-CGACTACGTGTACATTTTCCG as: 5’-GATTCCGATGTCTTTGGCTC	445
Mouse Cftr NM_021050.2	s: 5′-GAATCCCCAGCTTATCCACG as: 5′-CTTCACCATCATCTTCCCTAG	544
Human CFTR NM_000492.4	s: 5’-CGACTACGTGTACATTTTCCG as: 5′-GCTCTTGTGGACAGTAATATATCG	568
Human CLCA1 NM_001285.4	s: 5′-GGGGCCATTTAAGAGTTCTG as: 5′-CTCTCCACAGTTGCCCATC	379
Mouse Slc26a9 NM_177243.4	s: 5′-CATTTGCTGCGCTCTCTCAG as: 5′-CCTCTTCTCCTGCTTCCGG	568
Mouse Kcnn4 NM_008433.4	s: 5′-GAATCAGCCACAGTGTGTC as: 5′-CCTCCTTTGTCTTATTGTGG	515
Human KCNN4 NM_002250.3	s: 5′-GATTTAGGGGCGCCGCTGAC as: 5’-CTTGCCCCACATGGTGCCC	405
Human KCNQ1 NM_000218.3	s: 5′-CCACGGGGACTCTCTTCTG as: 5′-GGCACCTTGTCCCCATAG	505
Mouse Muc5ac NM_010844.3	s: 5′-CACCAAAGACAGCAGATCATC as: 5’-GTTCTGAGGACTCTGCATGG	295
Human SPDEF NM_012391.3	s: 5′-GTGCTCAAGGACATCGAGAC as: 5’-CCTAATGAAGCGGCCATAGC	423
Human Mouse Gapdh NM_001289726	s: 5′-GTATTGGGCGCCTGGTCAC as: 5′-CTCCTGGAAGATGGTGATGG	200

## Data Availability

Not applicable.

## Supplementary Materials

The following are available online at https://www.mdpi.com/article/10.3390/ijms22105133/s1, Supplementary Figure S1. CLCA1 produced in HEK293 cells, Figure S2: Upregulation of KCNN4 channels in asthma, Supplementary Figure S3. Activation of TMEM16A and KCNN4 in 6CFSMEo- human submucosal glands, Supplementary Figure S4. Activation of whole cell currents by N-CLCA1.
