# Supplementary material for: CLCA1 Regulates Airway Mucus Production and Ion Secretion Through TMEM16A
Source: Int J Mol Sci. 2021 May 12;22(10):5133. doi: 10.3390/ijms22105133 (PMC8151571; doi:10.3390/ijms22105133)
Supplement: Supplementary file 1 [file ijms-22-05133-s001.zip › ijms-1199453-supplementary.pdf]

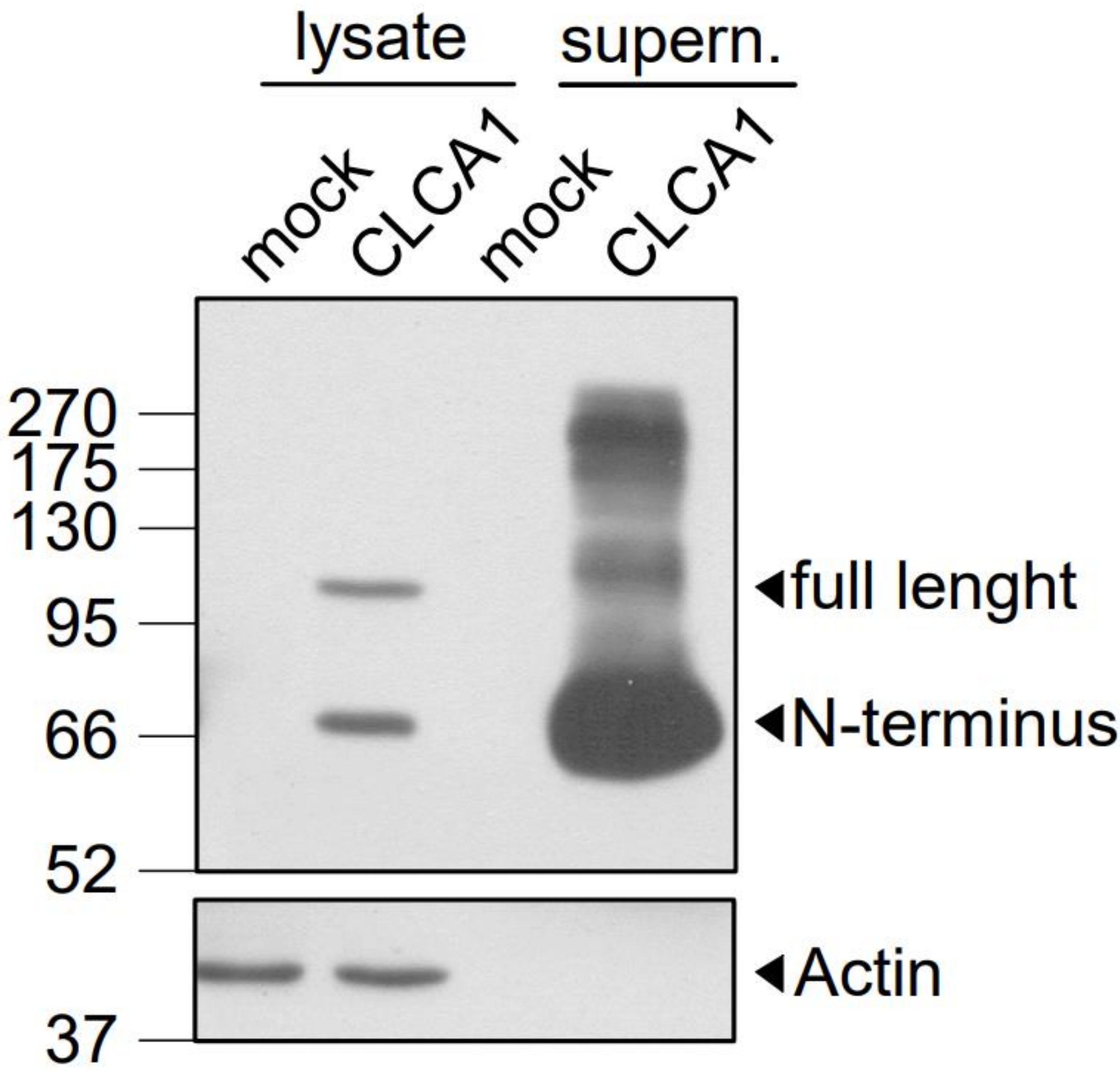

Figure S1. CLCA1 produced in HEK293 cells. Cells were transfected with human CLCA1 plasmid and secreted CLCA1 was harvested in the supernatant. Western blot of full length CLCA1 and secreted CLCA1-N terminus (N-CLCA1) harvested in the supernatant.

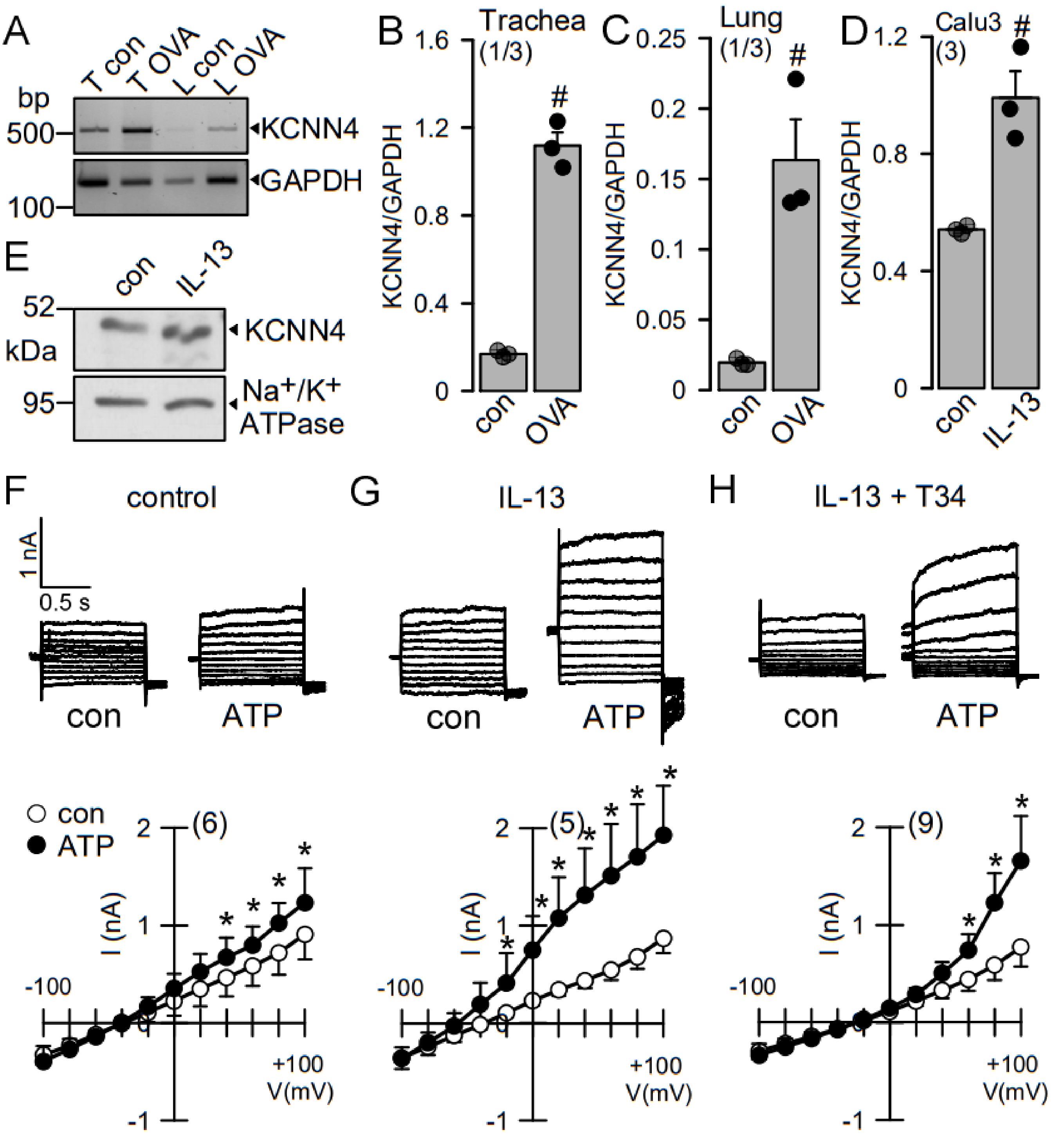

Figure S2. Upregulation of KCNN4 channels in asthma. (**A**–**C**) Original blot and summary for sqRT PCR indicating upregulation of expression of the Ca^{2+}-activated K^+ channel KCNN4 in trachea (T) and lungs (L) of OVA-treated asthmatic mice. (**D**) Summary of sqRT-PCR indicating upregulation of expression of KCNN4 in Calu3 airway epithelial cells after incubation with IL-13 (20 ng/mL). (**E**) Western blot of KCNN4 expressed in Calu3 cells suggesting slight upregulation by IL-13. (**F**–**H**) Whole cell patch clamp overlay currents and current/voltage relationships in Calu3 cells under control conditions, in the presence of IL-13, and in the presence of IL-13 and the KCNN4 blocker TRAM-34 (T34, 100 nM). ATP (100 µM) activated a whole cell current and hyperpolarized the membrane voltage in cells exposed to IL-13, suggesting predominant activation of KCNN4. In the presence of TRAM-34, whole cell currents were still activated but the membrane voltage remained depolarized. Application of ATP in the presence of the KCNN4-inhibitor Tram-34 depolarized the membrane voltage, indicating predominant activation of TMEM16A. Mean ± SEM (number of experiments). # indicates significant difference when compared to the absence of OVA or IL-13 ($p < 0.05$; unpaired t-test). *indicates significant activation by ATP ($p < 0.05$; paired t-test).

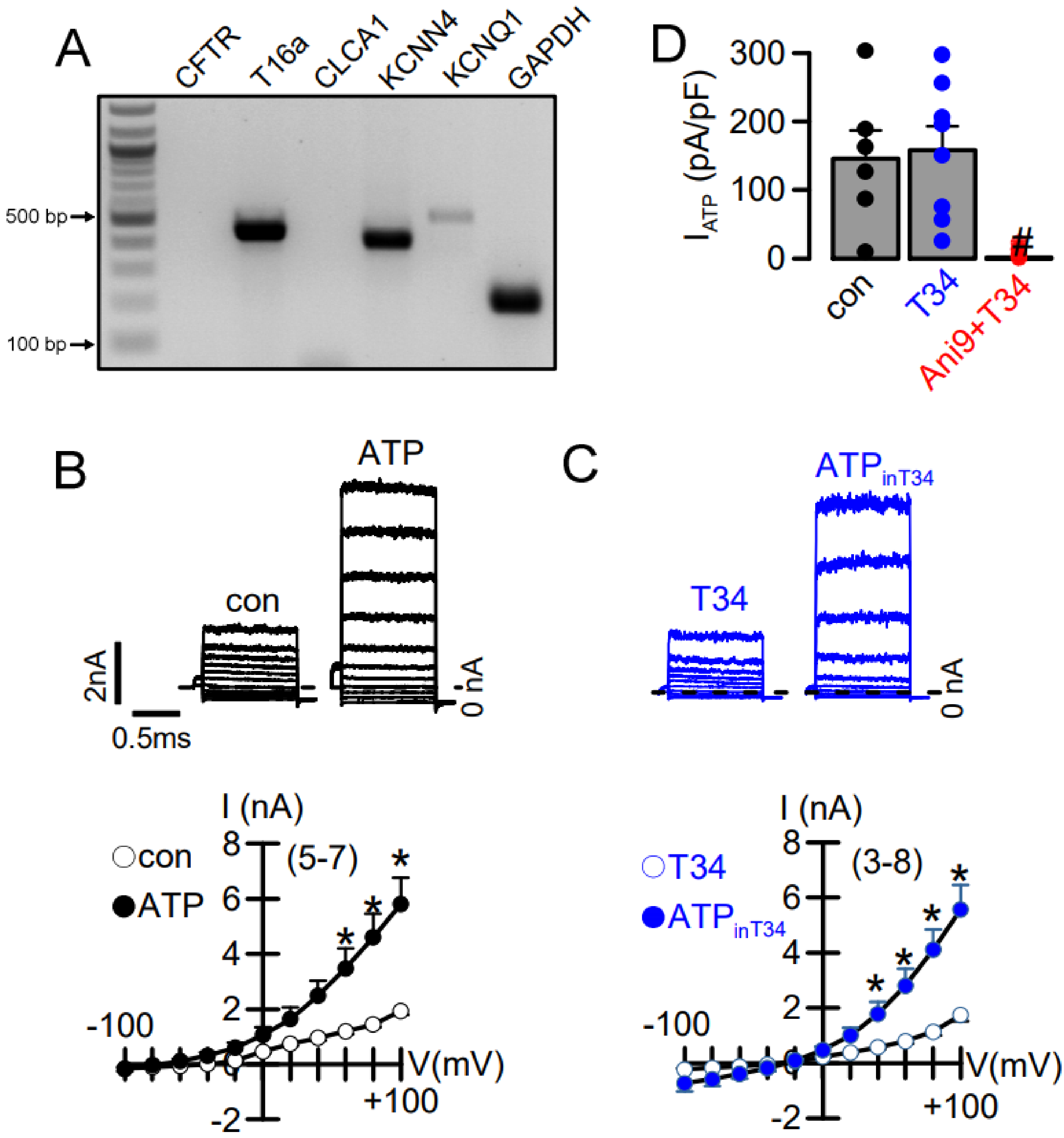

Figure S3. Activation of TMEM16A and KCNN4 in. 6CFSMEo- human submucosal glands. (**A**) RT-PCR indicating pronounced expression of Ca^{2+}-dependent TMEM16A and KCNN4, with little or no expression of CFTR, CLCA1, or KCNQ1. (**B,C**) Whole cell currents and current/voltage relationships activated by ATP (1 µM) in the absence or presence of the KCNN4-inhibitor Tram-34 (T34; 100 nM). In the absence of T34, cells were hyperpolarized by ATP, suggesting predominant activation of KCNN4 K^+ channels. In the presence of Tram-34, KCNN4 was blocked and ATP depolarized the cells by activation of TMEM16A. D) Summary of ATP-activated whole cell currents in the absence or presence of inhibitors. Mean ± SEM (number of experiments). $^{\#}$ indicates significant difference ($p < 0.05$; unpaired t-test). *indicates significant activation by ATP ($p < 0.05$; paired t-test).

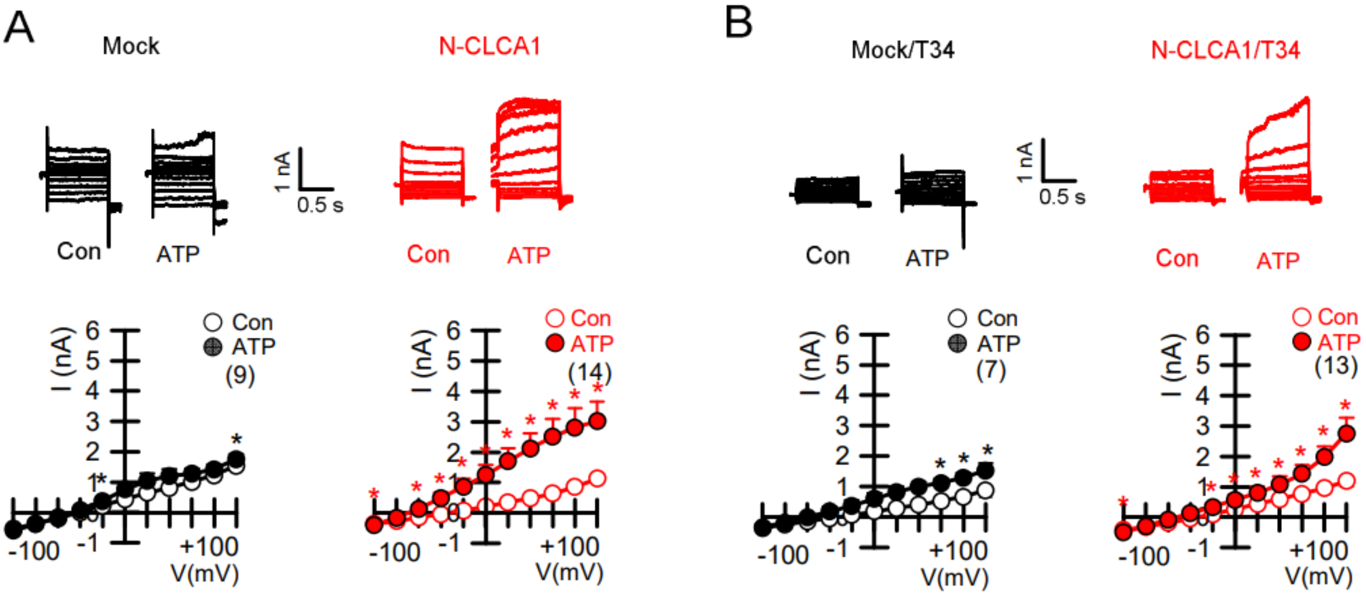

Figure S4. Activation of whole cell currents by N-CLCA1. Whole cell currents and current/voltage relationships in Calu3 cells under control conditions and after exposure to CLCA1 N-terminus (N-CLCA1) in the absence (**A**) or presence (**B**) of the KCNN4-inhibitor TRAM-34 (T34; 100 nM). ATP (100 µM) activated a whole cell current and hyperpolarized the membrane voltage after incubation of the cells with N-CLCA1, suggesting predominant activation of KCNN4 K^+ channels. In the presence of TRAM-34, KCNN4 currents were inhibited and ATP no longer hyperpolarized the cells. In the presence of TRAM-34, I/V relationships were more outwardly rectifying and peak currents at +100 mV were similar as in the absence of TRAM-34. The data indicate that upon increase in intracellular Ca^{2+}, Calu3 cells activate predominantly KCNN4 K^+ currents. Mean ± SEM (number of experiments). *indicates significant activation by ATP ($p < 0.05$; paired t-test).
